# Acinic cell carcinoma of the parotid gland: *Timeo Danaos et dona ferentes*? A multicenter retrospective analysis focusing on survival outcome

**DOI:** 10.1007/s00405-022-07481-w

**Published:** 2022-06-09

**Authors:** Pietro De Luca, Luca de Campora, Domenico Tassone, Francesca Atturo, Roberta Colangeli, Gerardo Petruzzi, Matteo Fermi, Giulia Molinari, Andi Abeshi, Giulia Cintoli, Alfredo Lo Manto, Giulia Togo, Filippo Ricciardiello, Paolo Condorelli, Ferdinando Raso, Arianna Di Stadio, Giovanni Salzano, Erik Esposito, Aurelio D’Ecclesia, Marco Radici, Maurizio Iemma, Maurizio Giovanni Vigili, Francesco Antonio Salzano, Luciano Magaldi, Michele Cassano, Iacopo Dallan, Raul Pellini, Livio Presutti, Franco Ionna, Enrico de Campora, Angelo Camaioni

**Affiliations:** 1 Department of Medicine, Surgery and Dentistry, University of Salerno, Salerno, Italy; 2 Department Otolaryngology Head and Neck Surgery, San Giovanni-Addolorata Hospital, Rome, Italy; 3 Department Otolaryngology Head and Neck Surgery, IRCCS Regina Elena National Cancer Institute, Istituti Fisioterapici Ospitalieri (IFO), Rome, Italy; 4 Department of Otorhinolaryngology-Head and Neck Surgery, IRCCS Azienda Ospedaliero-Universitaria di Bologna, Bologna, Italy; 5 Department of Specialist, Diagnostic and Experimental Medicine, Alma Mater Studiorum University, Bologna, Italy; 6 Department of Otolaryngology-Head and Neck Surgery, University of Foggia, Foggia, Italy; 7 Department of Otorhinolaryngology-Head and Neck Surgery, University Hospital of Modena, Modena, Italy; 8 Maxillofacial Surgery Unit, Department of Neurosciences, Reproductive and Odontostomatological Sciences, University Federico II, Naples, Italy; 9 Otolaryngology Department, AORN Cardarelli, Naples, Italy; 10 Otolaryngology Department, AORN Garibaldi, Catania, Italy; 11 Otolaryngology Department, University of Catania, Catania, Italy; 12 Otolaryngology and Maxillo-Facial Surgery Unit, Istituto Nazionale Tumori-IRCCS Fondazione G. Pascale, Naples, Italy; 13 Otolaryngology Department, ASL Napoli 3 Sud, Torre del Greco, Naples, Italy; 14 IRCCS “Casa Sollievo Della Sofferenza” San Giovanni Rotondo, Foggia, Italy; 15 Unit of Otolaryngology, S. Giovanni Calibita-Fatebenefratelli General Hospital, Rome, Italy; 16 Otolaryngology Department, San Giovanni di Dio e Ruggi D’Aragona University Hospital, Salerno, Italy; 17 Department of General Surgery-Head and Neck Consultant, Istituto Dermopatico Dell’Immacolata IDI-IRCCS, Rome, Italy; 18 Department of Otorhinolaryngology, Azienda Ospedaliera Universitaria, Pisa, Italy; 19 Associazione Ospedaliera Italia Centromeridionale Otorinolaringoiatrica (AOICO), Rome, Italy

**Keywords:** Acinic cell carcinoma, Parotid gland, Major salivary gland tumour, Parotid cancer

## Abstract

**Objectives:**

To analyze the demographic data, surgical and adjuvant treatment data and the survival outcomes in adult patients affected by acinic cell carcinoma of the parotid gland (AciCC).

**Methods:**

A retrospective multicenter analysis of patients treated for AciCC of the parotid gland from 2000 to 2021 was performed. Exclusion criteria were pediatric (0–18 years) patients, the absence of follow-up and patients with secondary metastatic disease to the parotid gland. Multivariable logistic regression was used to determine factors associated with survival.

**Results:**

The study included 81 adult patients with AciCC of the parotid gland. The median age was 46.3 years (SD 15.81, range 19–84 years), with a gender female prevalence (*F* = 48, *M* = 33). The mean follow-up was 77.7 months (min 4–max 361, SD 72.46). The 5 years overall survival (OS) was 97.5%. The 5 years disease-free survival (DFS) was 60%. No statistical differences have been found in prognosis for age (< 65 or ≥ 65 years), sex, surgery type (superficial vs profound parotid surgery), radicality (R0 vs R1 + Rclose), neck dissection, early pathologic T and N stages and adjuvant therapy (*p* > 0.05).

**Conclusion:**

This study did not find prognostic factor for poorest outcome. In contrast with the existing literature, our results showed how also high-grade tumours cannot be considered predictive of recurrence or aggressive behaviour.

**Supplementary Information:**

The online version contains supplementary material available at 10.1007/s00405-022-07481-w.

## Introduction

Acinic cell carcinoma (AciCC) is the fourth most common epithelial tumour of salivary glands after mucoepidermoid carcinoma, adenocarcinoma (not otherwise specified, NOS) and adenoid cystic carcinoma [1]; it can be considered an extremely rare tumour (it comprises 9–11% of all adult parotid malignancies) [2] and its incidence has been previously described by the Surveillance of Rare Cancers in Europe Working Group (RARECARE) as 1.20–1.63 cases per 1,000,000 patients/years [3]. The definition of AciCC is constantly evolving over time; first observed in 1892 from Nasse [4] as a benign lesion and called “adenoma”, it was described by Buxton et al. [5] as a malignant tumour with the ability to metastasize and recur. Finally, it was defined as AciCC by Godwin et al. in 1954 [6]. According to the latest histological classification of the World Health Organization (WHO), AciCC is defined as “a malignant epithelial neoplasm of salivary glands in which at least some of the neoplastic cells demonstrate serous acinar cell differentiation, which is characterized by cytoplasmic zymogen secretory granules. Salivary ductal cells are also a component of this neoplasm” [2]. Despite it having been considered for decades a low-grade indolent tumour with a good prognosis, its propensity to recur and metastasize, especially for a subset of lesions with high-grade transformation, currently, it is quite common to consider AciCC as a malignancy of uncertain course [7]. In addition, there is a great variability in studies about treatment recommendations and prognosis; surgical excision is the recommended option to manage AciCC, while the use of radiotherapy is still controverse, even in AciCC with close margins.

The aim of this retrospective study is to present a multicenter experience in the treatment of AciCC, focusing on the demographic, surgical and adjuvant treatment data and the survival outcomes in patients affected by AciCC. To the best of our knowledge, this is the largest European case series and those with the longest follow-up reporting experience about this rare parotid tumour.

## Materials and methods

### Objectives

The primary endpoint was to assess the survival outcomes (progression-free survival, PFS, from diagnosis to first progression; overall survival, OS) of patients treated with curative intent for AciCC of parotid gland. The secondary endpoints were to evaluate the presence of prognostic factors and to describe the progression sites.

### Patients

For this multicenter analysis, patients with biopsy proven AciCC of the parotid gland were retrospectively included, treated between January 2000 and September 2021. Patients received surgery with curative intent at the Departments of Otolaryngology-Head and Neck Surgery of San Giovanni-Addolorata Hospital, Rome, Italy; San Giovanni Di Dio e Ruggi D’Aragona Hospital, Salerno, Italy; University of Modena, Modena, Italy; University of Bologna, Bologna, Italy; IRCCS Regina Elena National Cancer Institute, Rome, Italy; AORN Cardarelli, Napoli, Italy; IRCCS Fondazione G. Pascale, Napoli, Italy; University of Foggia, Foggia, Italy; ASL Napoli 3 Sud, Napoli, Italy; AORN Garibaldi, Catania, Italy; San Giovanni Calibita-Fatebenefratelli General Hospital, Rome, Italy; IRCCS “Casa sollievo della sofferenza” San Giovanni Rotondo, Foggia, Italy; San Carlo di Nancy Hospital, Rome, Italy; University of Pisa; IRCCS Fondazione G. Pascale, Naples, Italy. All the Departments involved in this study are part of *Associazione Ospedaliera Italia Centromeridionale Otorinolaringoiatrica* (AOICO; www.aoico.it). Exclusion criteria were pediatric (0–18 years) patients, the absence of follow-up and patients with secondary metastatic disease to the parotid gland.

### Demographics, clinical, surgical and pathological data

Data concerning gender, age at the time of diagnosis, fine-needle aspiration biopsy (FNAB) diagnosis, type of surgery upfront (including eventual neck dissection at the time of first surgery), lymph node or distant metastasis at the time of diagnosis, post-operative resection margins and pTNM, adjuvant radiochemotherapy, locoregional or distant recurrences and the status at the follow-up (including the time of follow-up, months) were systematically collected. The data concerning survival and recurrence outcomes were retrieved from mortality registries, outpatient visits and radiological follow-up. All histologic slides were reviewed from pathologists of each department to confirm the diagnosis of AciCC of the parotid gland. The data about histologic grading were obtained only from recurrent or metastatic AciCC.

### Ethical considerations

Ethical approval was waived by the local ethics committee in view of the retrospective nature of the study. The study was conducted in compliance with the Helsinki Declaration. All the clinical data needed for the study were recorded in a computerized database. Patients who were alive at study time were informed about the study and none expressed opposition to inclusion.

### Statistical analysis

Quantitative variables were described with median and range [min–max], while qualitative variables were described with numbers and percentages. Chi square or Fisher exact tests were used to compare categorical variables. PFS and OS were assessed using the Kaplan–Meier estimator and compared using a log-rank test. For all tests, a two-tailed *p* value less than or equal to 0.05 was considered statistically significant. To identify factors independently associated with decreased OS, a univariate then multivariable Cox regression analysis was performed. Measures of precision of point estimates are presented as odds ratios or hazard ratios with 95% CI.

## Results

### Population

Patients with diagnosis of AciCC of the parotid gland in the period study have been 94. Thirteen patients were excluded because of the age (pediatric patients, *n* = 7) or because of loss to follow-up (*n* = 6). Therefore, the study group included 81 adult patients with AciCC. The median age was 46.3 years (SD 15.81, range 19–84 years), with a gender female prevalence (*F* = 48, *M* = 33). The incidence showed a peak in the fifth decade (*n* = 23, 28.4%), with the most of the cases (*n* = 54, 66.7%) occurring in the fourth, fifth and sixth decades. Pre-operative evidence of cervical node involvement (cN +) was identified in 7.4% (*n* = 6) of cases. Detailed patient demographics and oncologic data are summarized in Table 1.

**Table 1:** Characteristics of the cohort of patients with AciCC of the parotid gland

Variable		*N*	%
Participants		81	
Mean age (min–max–SD)	46.3 years (19–84–15.81)		
Gender	Female	48	59.2
	Male	33	40.7
FNAB result	Not diagnostic	43	53.1
	Other parotid neoplasm	23	28.4
	Acinic cell carcinoma	11	13.5
	Not performed	4	4
Parotid surgery	I–IV (VII) [Total parotidectomy with facial nerve resection]	1	1.2
(According to European Salivary	I–IV [Total parotidectomy]	34	41.9
Gland Society Classification)	I–II [Superficial parotidectomy]	41	50.6
	I–II–III [Superficial parotidectomy extended to inferior deep lobe]	5	6.2
Nodal dissection (ND)	No ND performed	66	81.5
	ND performed	15	18.5
	Selective (II–IV)	11	73.3
	Superselective (II or II–III)	3	20
	mRND/RND (I–V)	1	6.7
Margin status	R0	71	87.6
	R1	7	8.6
	Rclose	3	3.7
Staging	Stage I	59	72.8
	Stage II	1	1.2
	Stage III	14	17.3
	Stage IVA	7	8.6
Adjuvant treatment	No	60	74.1
	Yes (Radiotherapy)	21	25.9
Pathologic T classification (according to TNM classification- 8th edition)	T1	32	39.5
	T2	35	43.2
	T3	11	13.5
	T4a	3	3.7
Pathologic N classification (according to TNM classification-8th edition)	N0	72	88.9
	N1	4	4.9
	N2a	2	2.5
	N2b	3	3.7
Recurrence	Local	9	11.5
	Distant metastasis	1	1.4
Mean follow-up (min, max)	54.5 (4–361, months		
Status at last follow-up	NED	66	81.5
	DOOC	6	7.4
	NED II	4	4.9
	DOD	3	3.7
	AWD	2	2.5

*SD* standard deviation, *FNAB* fine-needle aspiration biopsy, *DNA* data not available, *ND* neck dissection, *NED* no evidence of disease, *NED II* no evidence of disease after recurrence, *AWD* alive with disease, *DOD* died of disease, *DOOC* died of other cause

Pre-operative FNAB was performed in 95.1% (*n* = 77) of cases and resulted positive for AciCC only in 14.3% of the cases (*n* = 11). FNAB was not diagnostic in 55.8% of the patients (*n* = 43) and other parotid malignancies were shown in 23 cases (29.9%). In this study, none of the patients showed distant metastasis at the time of initial diagnosis of AciCC.

### Treatment characteristics

All patients were treated with surgery; the performed surgical procedure depended on the tumour location and the extent of disease. According to European Salivary Glands Society (ESGS) classification of parotidectomies [8], the 50.6% of the patients (*n* = 41) were treated with superficial parotidectomy (I–II), 41.9% (*n* = 34) with total parotidectomy (I–IV), 6.2% (*n* = 5) with superficial parotidectomy extended to inferior deep lobe (I–II–III) and 1.2% (*n* = 1) with total parotidectomy with facial nerve resection (I–IV [VII]). Neck dissection (ND) was not planned in 81.5% (*n* = 66) of the cases, while 18.5% (*n* = 15) received ND; in this group of patients, 73.3% (*n* = 11) received selective ND (levels II–IV).

Among the 81 patients included, 21 (25.9%) received adjuvant radiotherapy (RT) after surgery; among these 21 patients, 14 (66.7%) received locoregional irradiation despite not having positive cervical lymph nodes. In addition, 9 patients (60%) among those received ND (*n* = 15), underwent adjuvant radiotherapy. In this study, none of the patients received chemotherapy as adjuvant post-operative therapy.

### Staging

The definitive staging of disease is shown in Table 1. Most patients showed no positive cervical nodal metastases. Post-operative N staging was positive in 9 patients (11.1%). The majority of cases were classified as pN1 (*n* = 4, 4.9%); other pN + (pN2a or pN2b) were rarely observed (*n* = 2, 2.5%, for pN2a and *n* = 3, 3.7%, for pN2b). No pN2c (bilateral cervical nodal metastases) nor pN3a category (nodal metastases with the largest diameter > 60 mm) were observed in this cohort of patients. Histologic grade was recovered only for recurrent or metastatic AciCC; seven of those patients showed low-grade tumour (77.8%), while only two subjects showed high-grade tumour (22.2%).

### Survival outcomes

**Fig. 1:**
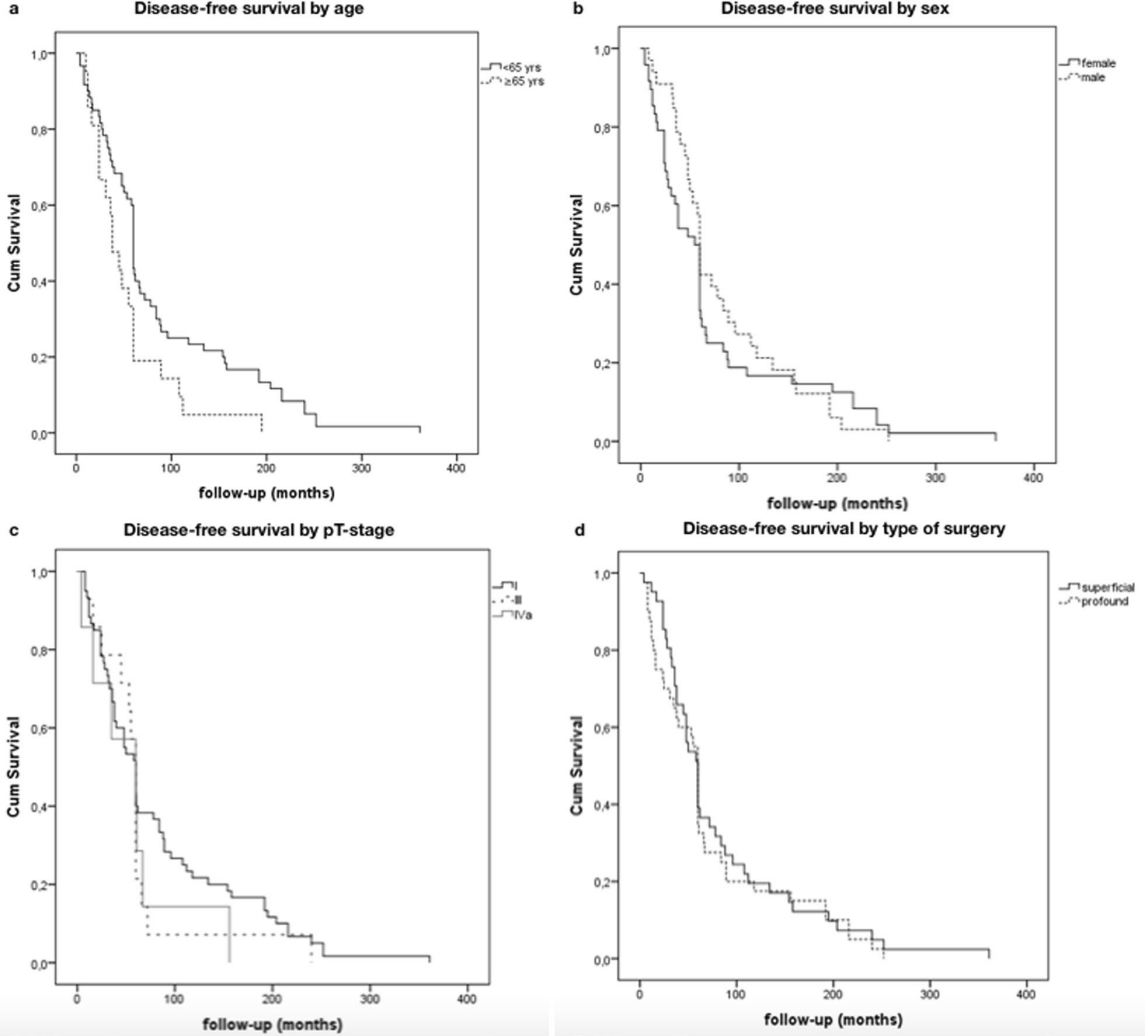
Kaplan–Meier analysis of 5-year and 10-year DFS of different variables. **a** Patients with age > 65 years (*n* = 21) had no a significantly decreased DFS when compared with those with age < 65 years (*n* = 60). **b** Male patients (*n* = 33) had no a significantly decreased DFS when compared with female patients (*n* = 48). **c** Patients with early pathologic T stages tumour (T1, *n* = 29; T2, *n* = 38) had no a significantly improved DFS compared with patients with late pathologic T stages disease (T3, *n* = 11; T4, *n* = 3). **d** Patients who underwent superficial parotidectomy (*n* = 41) had no a significantly improved DFS compared with patients who underwent total parotidectomy (*n* = 40)

Follow-up data were available for all the patients included in this study. Mean follow-up was 77.7 months (min 4–max 361, SD 72.46)]. The 5 years overall survival (OS) was 97.5% (95% CI 96.9–98) (Fig. 1a). The 5 years disease-free survival (DFS) was 60% (95% CI 56.1–63.9) (Fig. 1b).

**Fig. 2:**
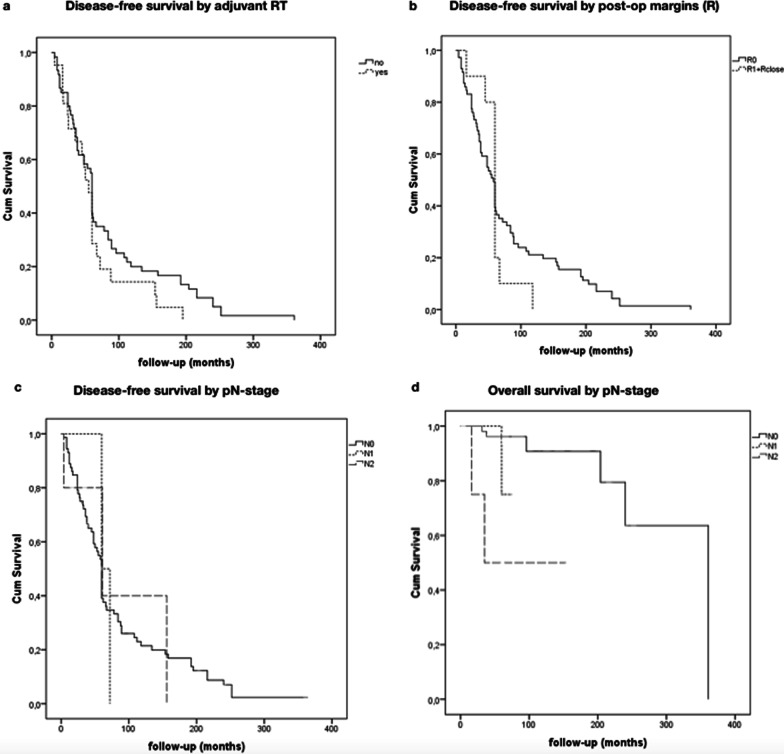
Kaplan–Meier analysis of 5-year and 10-year DFS of different variables. **a** Patients who underwent adjuvant radiotherapy (RT) (*n* = 21) had no a significantly improved DFS compared with patients who did not receive adjuvant RT (*n* = 60). **b** Patients with R0 post-operative margins (*n* = 71) had no a significantly improved DFS compared with patients with R1/Rclose margins (*n* = 10). **c** Patients with early pathologic N stages (*n* = 73) had no a significantly improved DFS compared with late pathologic N stages disease (N1, *n* = 3; N2, *n* = 5). **d** Patients with early pathologic N stages (*n* = 73) had a significantly improved OS compared with late pathologic N stages disease (N1, *n* = 3; N2, *n* = 5)

At the end of the study (October 2021), 88.9% patients (*n* = 72) were alive without any evidence of disease, 7.4% (*n* = 6) were alive, after recurrence, without any evidence of disease and 2.5% (*n* = 2) were alive with recurrence. Disease recurrence was found in 11.1% of the patients (*n* = 9), with a median interval to first recurrence of 53.1 months (SD 69.2, range 4–361 months). Distant recurrences were observed only in one patient (1.2%) and lung was the only organ involved. More patients died because of other cause (*n* = 6, 7.7%) than disease (*n* = 3, 3.8%). Survival data and univariate analysis of most relevant prognostic factors are shown in Fig. 2.

### Prognostic factors for survival

No statistical differences have been found in prognosis for age (< 65 or ≥ 65 years), sex, surgery type (superficial vs profound parotid surgery), radicality (R0 vs R1 + Rclose), neck dissection and adjuvant therapy (*p* > 0.05). Although the analysis of the Kaplan–Meier curves of DFS seems to suggest better results in T1 than T3 and T4, no significant difference has been found. The same for analysis of margin (R), where R0 seems to have better prognosis than R1and Rclose, and for age, where young patients seem to have better prognosis (Fig. 2). Multivariate analysis showed how recurrences or metastases can be considered a risk factor for worst status at the follow-up, while both the presence of recurrences of metastases and age > 65 years are independent prognosticators for worst outcome in terms of months of survival.

## Discussion

In this retrospective-multicenter study of patients diagnosed and treated for AciCC of the parotid gland, we investigated the demographic data, the presence of prognostic factors and the survival outcomes. To the best of our knowledge, this is the largest European surgical case series ever reported.

Despite AciCC is still considered a neoplasm with a good prognosis, several adverse features have been identified. In this study male patients, age above 65 years, advanced T stage and R1 and Rclose margins, seem to be associated with poorest survival, but without a statistical relevance; this data can support the suggestion by Scherl et al. [9], that an earlier diagnosis could play a prominent role in the progression of the tumours. Demographic data showed how women and 40–60 range patients were the most affected from the AciCC of the parotid gland. These results are in line with the previous literature [10]. The accuracy of FNAB diagnosis is very low and only 11 (13.5%) cases received the diagnosis of AciCC.

In this study, the rate of occult metastases was very low (2.5%), so this could suggest that elective neck dissection (END) should not be performed routinely; this is in accordance with the results from van Weert et al. [11], who found no occult metastases in END specimens from 89 patient with AciCC of the major salivary glands (not exclusively from parotid gland). On the contrary, Scherl et al. [9] found higher rate of occult metastases (22.3%), suggesting a protecting role of END in parotid AciCC. Also, Grasl et al. [12] suggested that END should be performed in patients with AciCC of the parotid gland, especially in patients with low-grade tumours, who showed a notable rate of occult neck metastases and worse DSS. Moon et al. [13] evaluated the risk of lymph node metastasis in a large cohort of patients with AciCC, concluding how this type of tumour carries a low risk of nodal metastasis, with a higher risk for advanced T stage (T3 and T4) and high-grade patients. According to our results, we cannot prove the definite benefit of END, also for patients with advanced T stage.

The indications for adjuvant RT in AciCC of the parotid gland are still debated; positive surgical margins, T3/T4 disease, high-grade tumours and lymphovascular/perineural invasion haven been considered as indications for post-operative RT [14], with no large case series supporting this evidence; anyhow, particularly in patients without any histopathologic risk factor, the exact indications are not well known. According to the results from Gomez et al. [15], who reported a cases series of 35 patients underwent surgery for AciCC of the parotid gland, a large number of patients had a low treatment failure rate and could be considered candidates for surgery only. Also, the work by Andreoli et al. [16] showed how RT do not provide a significant survival advantage for early-stage (T1 and T2) and lower-grade parotid AciCC. These conclusions are in accordance with those from a retrospective case series from Zenga et al. [17], in which patients with negative margins did not receive particular benefits from RT, due to a great loco regional control and surgical outcomes with surgery alone. Also, considering the potentially complications of neck RT (secondary malignancy, fistula, bone necrosis, xerostomia) [18, 19] and the lack of histological prognostic factors, the results of the present study suggest that post-operative RT should not be performed routinely.

The interpretation of this data is not easy due to impossibility to recover the specific indications for adjuvant therapy of each patient because of the retrospective nature of the study; however, our results seem to confirm the evidence of the previous works, showing no statistical significative difference between patients treated with post-operative RT and those not treated.

Different chemotherapy regimen has been proposed as adjuvant therapy, especially for metastatic disease [20], but none of these has been tried in a large randomized trial, due to the rarity of the neoplasm. In this study, only 1 patient (1.2%) underwent adjuvant chemotherapy, but we cannot provide information about the regimen therapy/monotherapy administered.

Recurrent and metastatic AciCC of the parotid gland are not uncommon, and there is no still consensus about the risk factors; in our work, nine patients experienced local recurrence, while only one patient experienced lung metastases. In our analysis, no risk factor can be identified as responsible of the progression of the disease.

AciCC with high-grade transformation is considered to be more aggressive and predictive of recurrence and distant metastases; despite a histologic grading system is still lacking, frequent mitosis, ki-67 proliferation > 5%, perineural and vascular invasion, infiltration and tumour necrosis are regarded as high-grade features, explaining the aggressive behaviour of these dedifferentiated tumours [21, 22]. The largest case series reported by Scherl et al. [9] found a subgroup of patients with high-grade features showing poor outcomes, suggesting how histologic grade can be a stronger predictor of surgical than T and N classification. In our study, in the group of nine patients with recurrent and/or metastatic AciCC of the parotid gland, only two of them showed high-grade transformation; for this reason, the results of the present work did not confirm the aggressive behaviour of high-grade AciCC, indeed they seemed to suggest that there is no association between the actual histologic grading and the poorer outcomes.

### Limits of the study

This study shows several limitations. First, this work included data prior to 2010, when mammary analogue secretory carcinoma (MASC), which exhibits a clinical behaviour similar to AciCC, was recognized as a distinct entity; anyhow, all histologic slides were reviewed from pathologists of each department to confirm the diagnosis of AciCC of the parotid gland. Second, we provided the data on a local level, from the Chief of each Head and Neck Department; however, to ensure a good level of quality control, the data were separately analyzed from two investigators (P.D.L., R.C.). Third, this is a retrospective study and the data lack about treatment decisions (surgery choice, management of the neck, selection of adjuvant therapy). Fourth, perivascular and perineurial invasion were not included in the evaluation; however, according to the last World Health Organization (WHO) Classification of Tumours, these two variables are currently not considered as independent prognostic factors. Despite these limits, this study represents the largest European cohort of patient affected by AciCC of the parotid gland and surgically treated with curative intent, and offers a notable analysis about the outcomes and the prognostic factors of this rare tumour. Furthermore, this study examined DSS and all follow-up information; in addition, this study analyzed the longest follow-up regarding AciCC of the parotid gland.

## Conclusion

We present here one of the largest case series ever reported about AciCC of the parotid gland, which represents the fourth parotid gland malignancy. AciCC commonly has a good prognosis, except for a little cohort of patients; in the present study univariate Cox analysis did not find any prognostic factor for poorest outcome. In contrast with the existing literature, our results showed how also high-grade tumours cannot be considered predictive of recurrence or aggressive behaviour. According to multilinear regression analysis, recurrences or metastases and age > 65 years can be independent prognosticators. The future goal of this study group will be the proposal of a new histologic grading to further stratify the patients, attempting to identify shared prognostic factors.

## Supplementary Information

Below is the link to the electronic supplementary material.

Supplementary file1 (DOCX 5209 KB)
