# Supplementary figures and images for: Acinic cell carcinoma of the parotid gland: Timeo Danaos et dona ferentes? A multicenter retrospective analysis focusing on survival outcome
Source: Eur Arch Otorhinolaryngol. 2022 Jun 9;279(12):5821–9. doi: 10.1007/s00405-022-07481-w (PMC9649501; doi:10.1007/s00405-022-07481-w)

Effect of the variables on the status follow-up


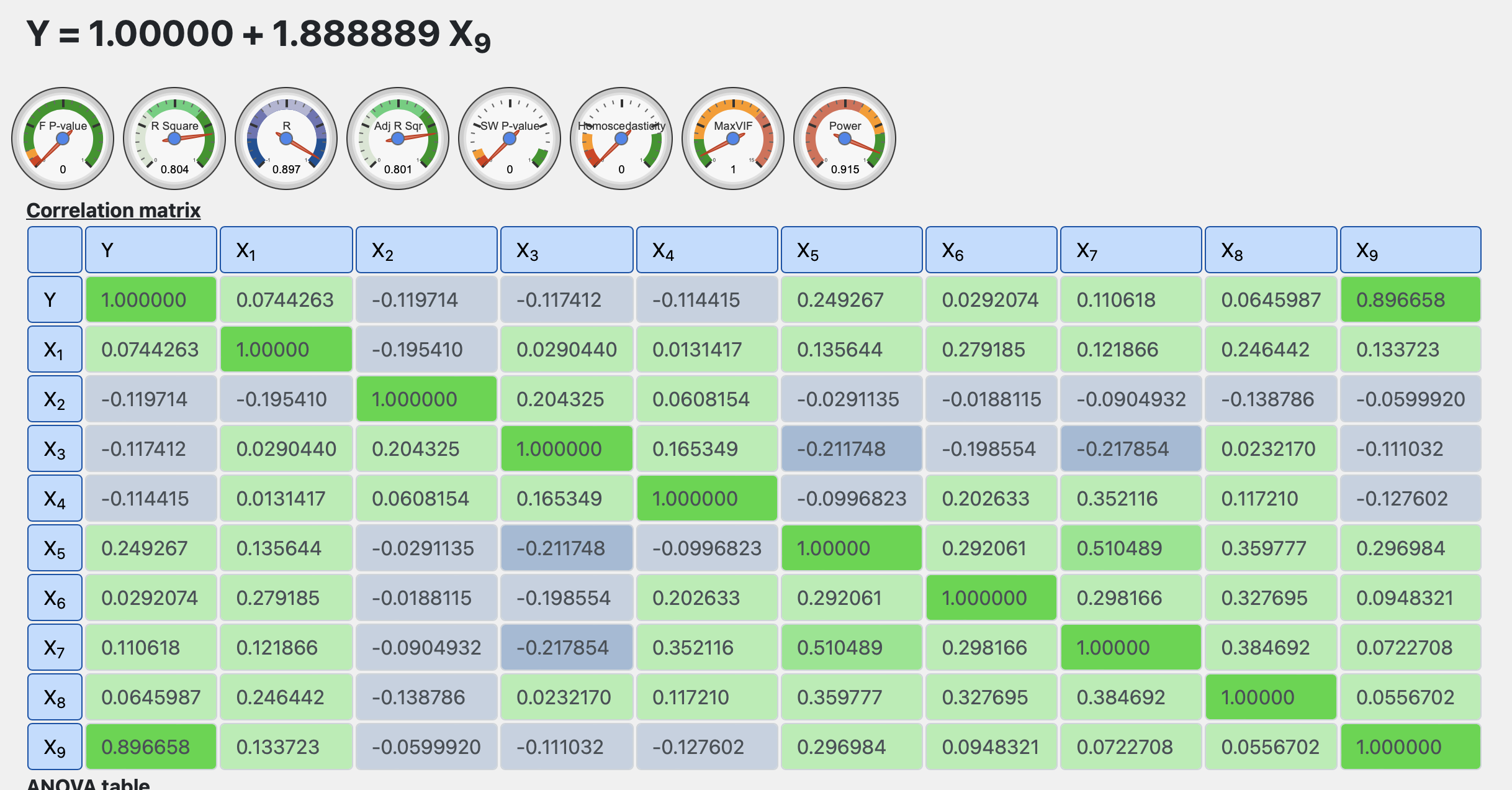


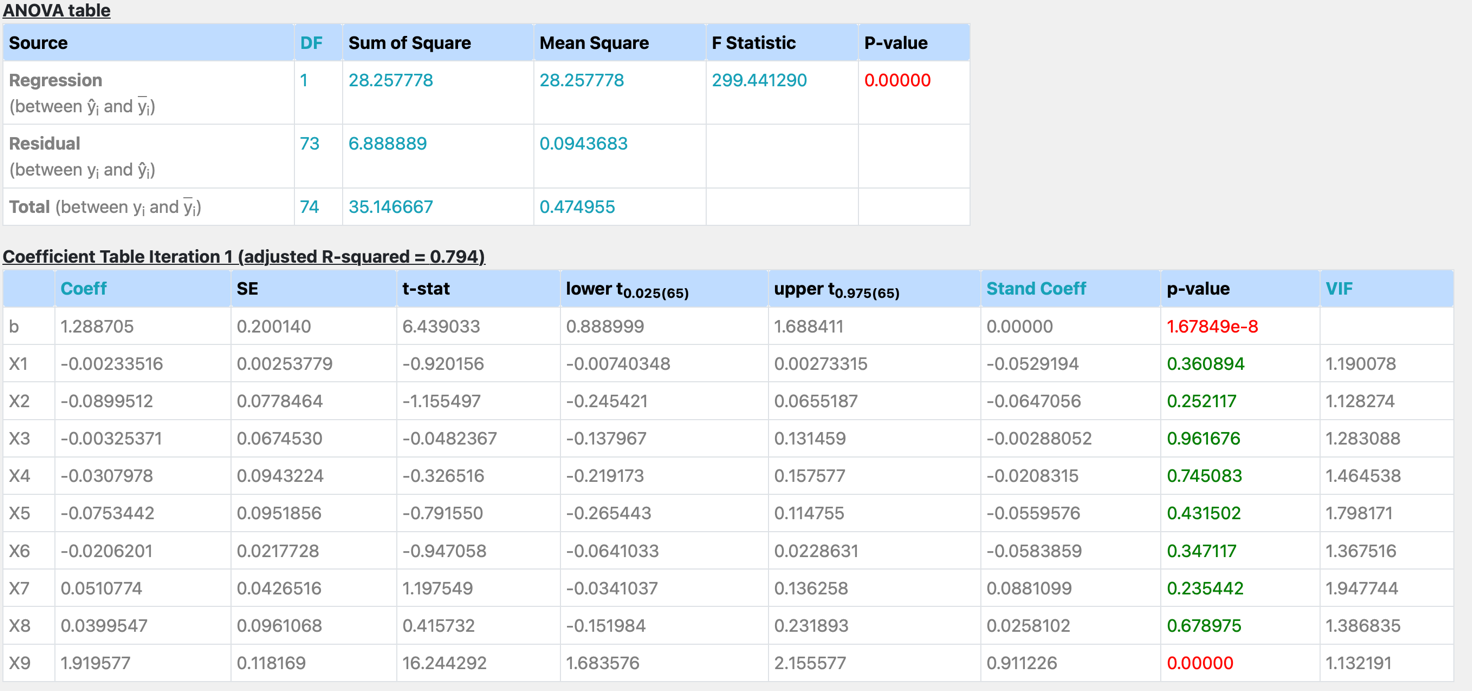


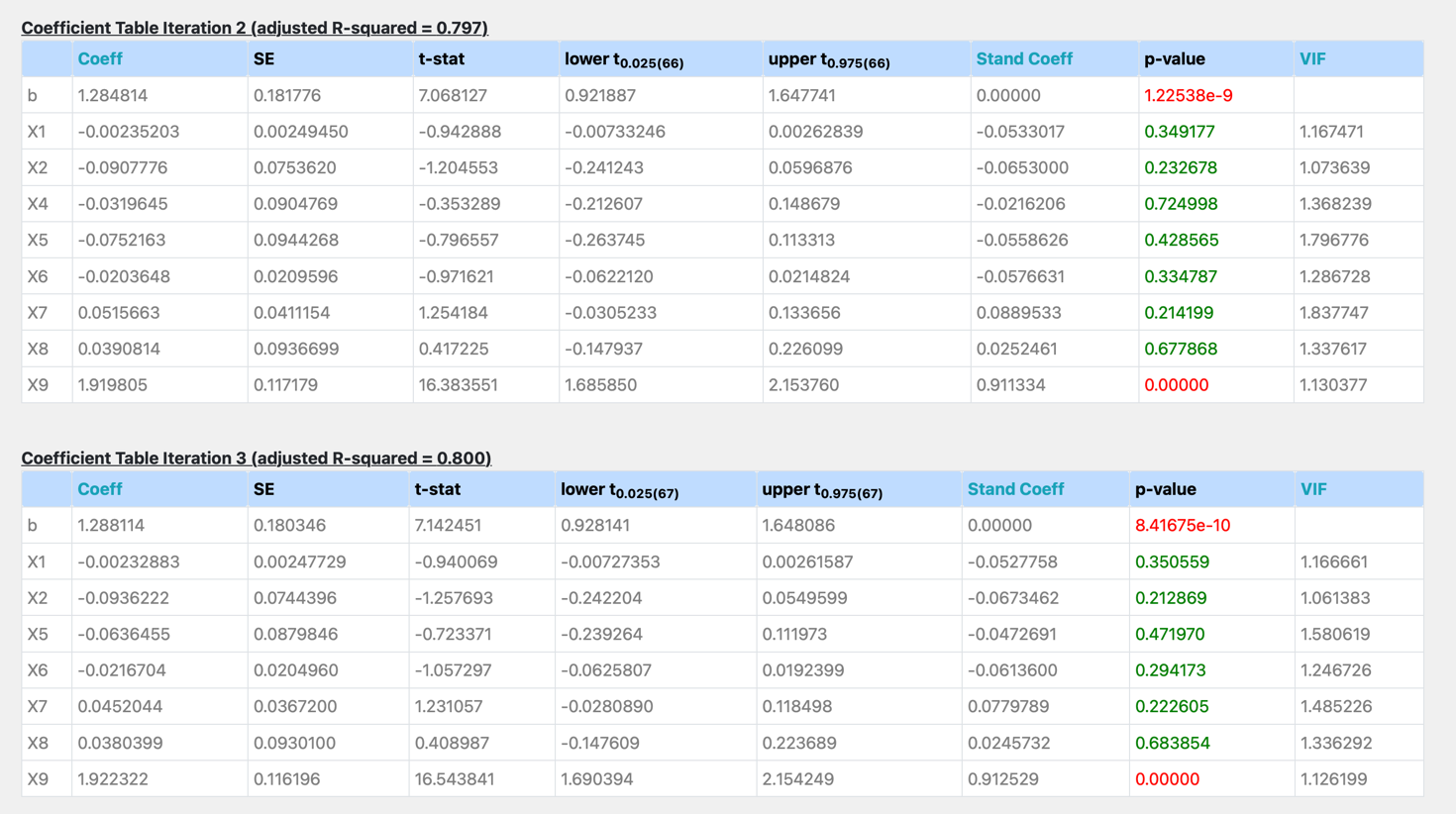


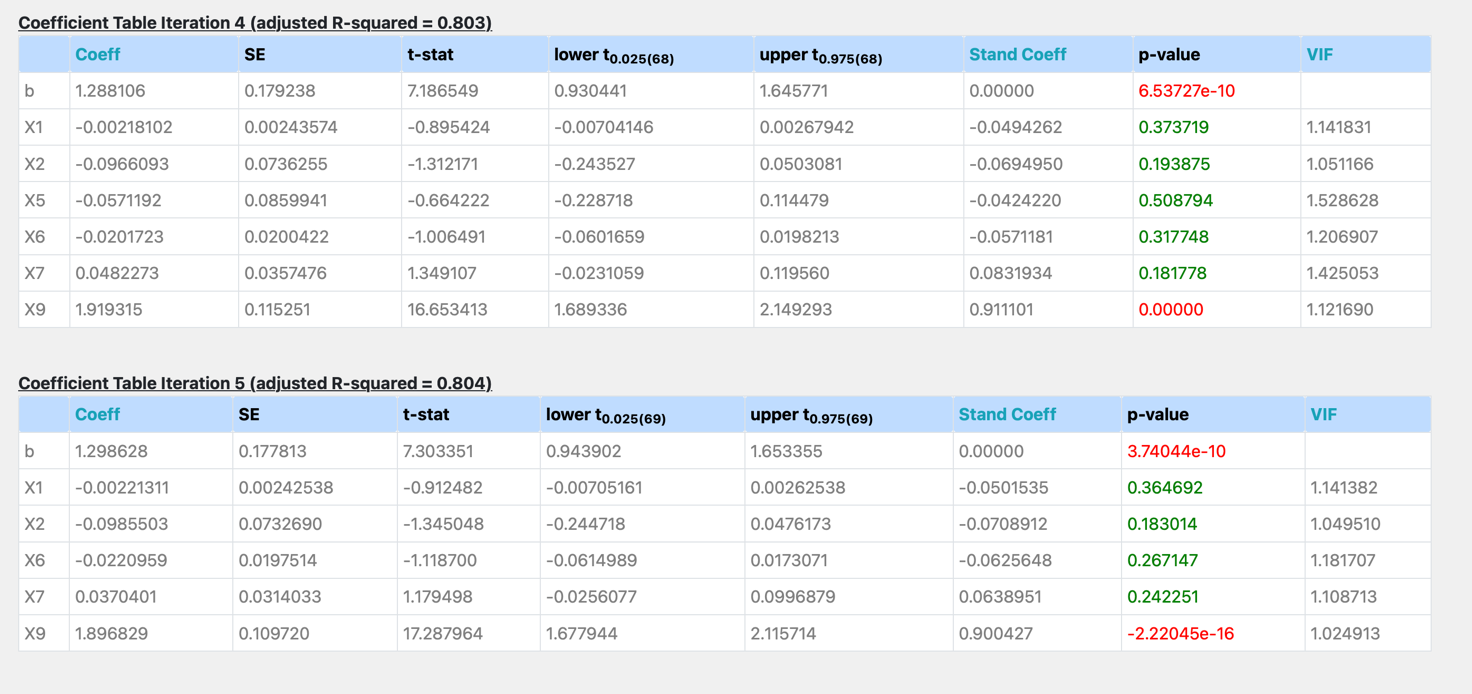


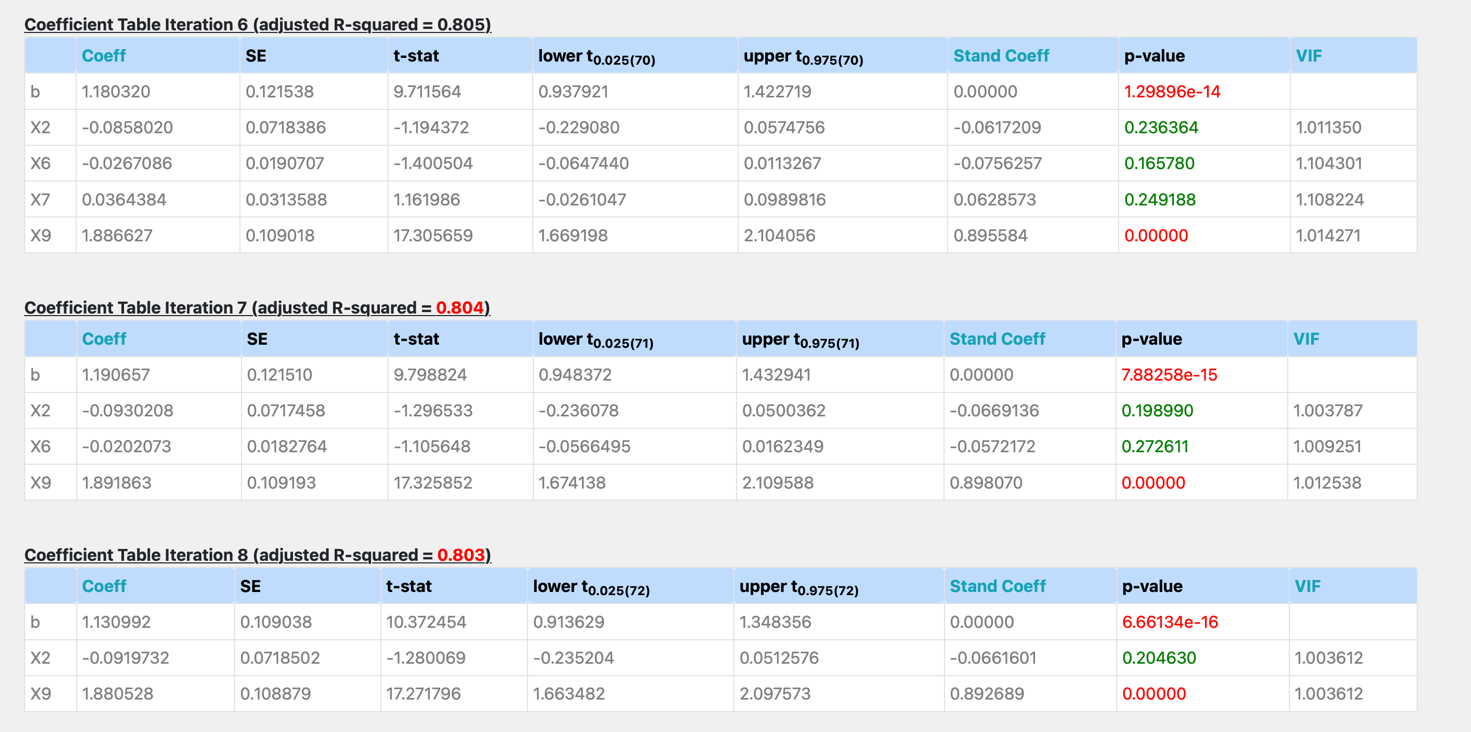


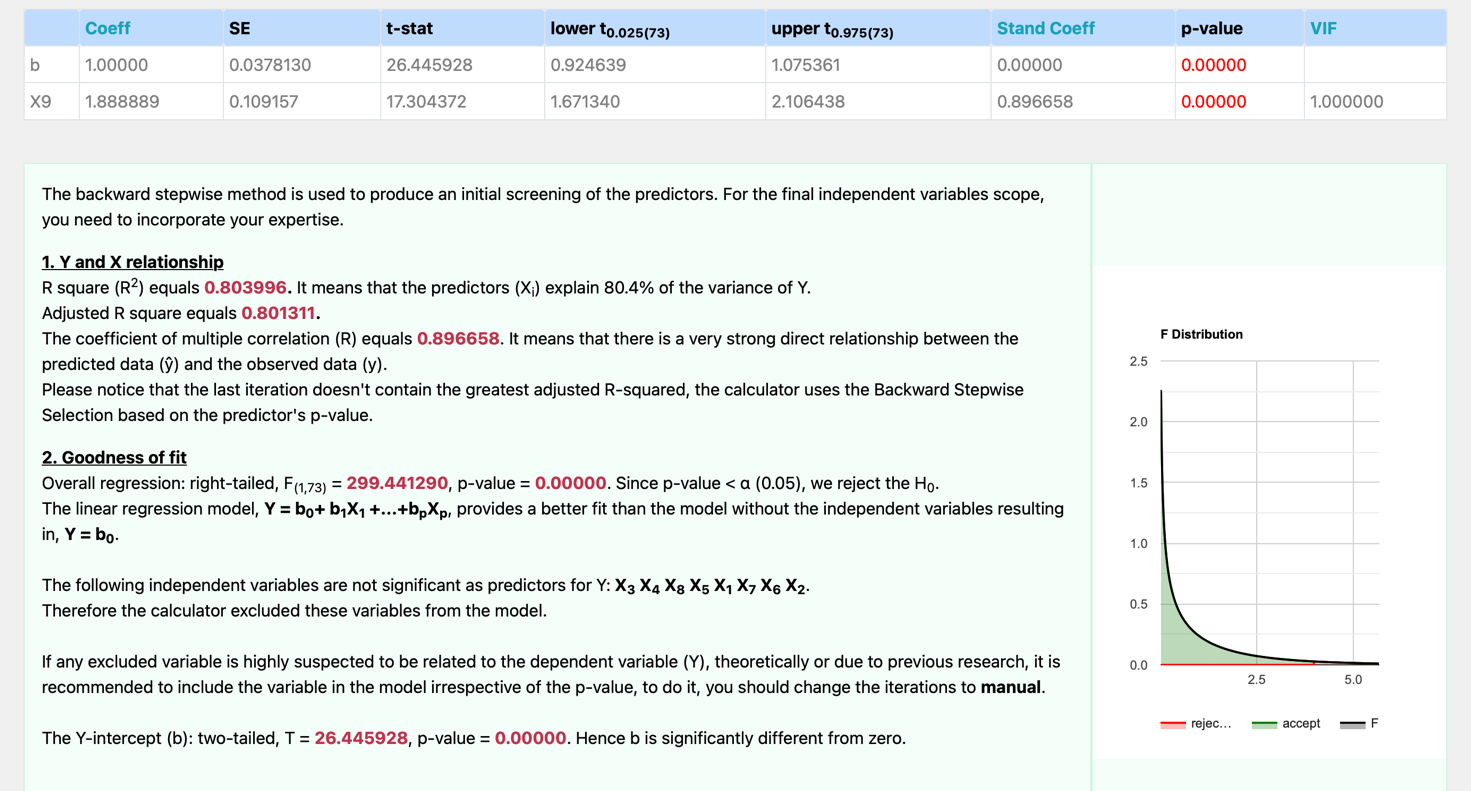


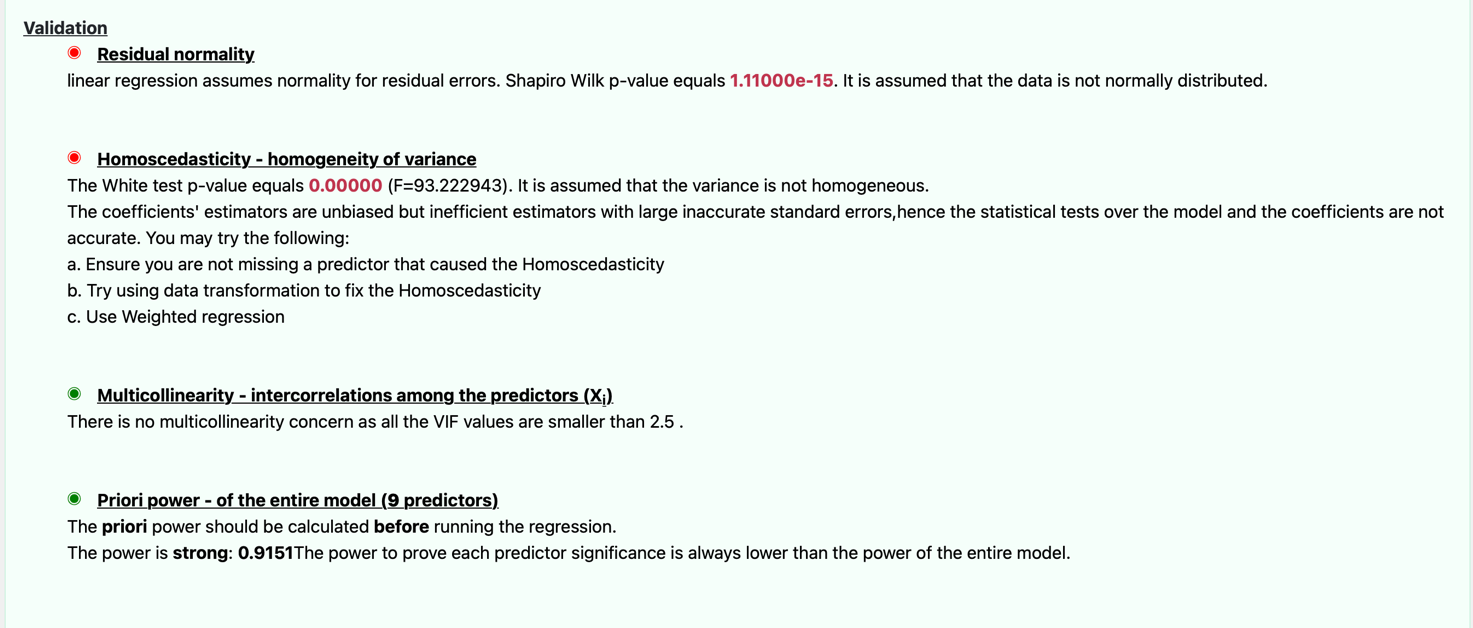


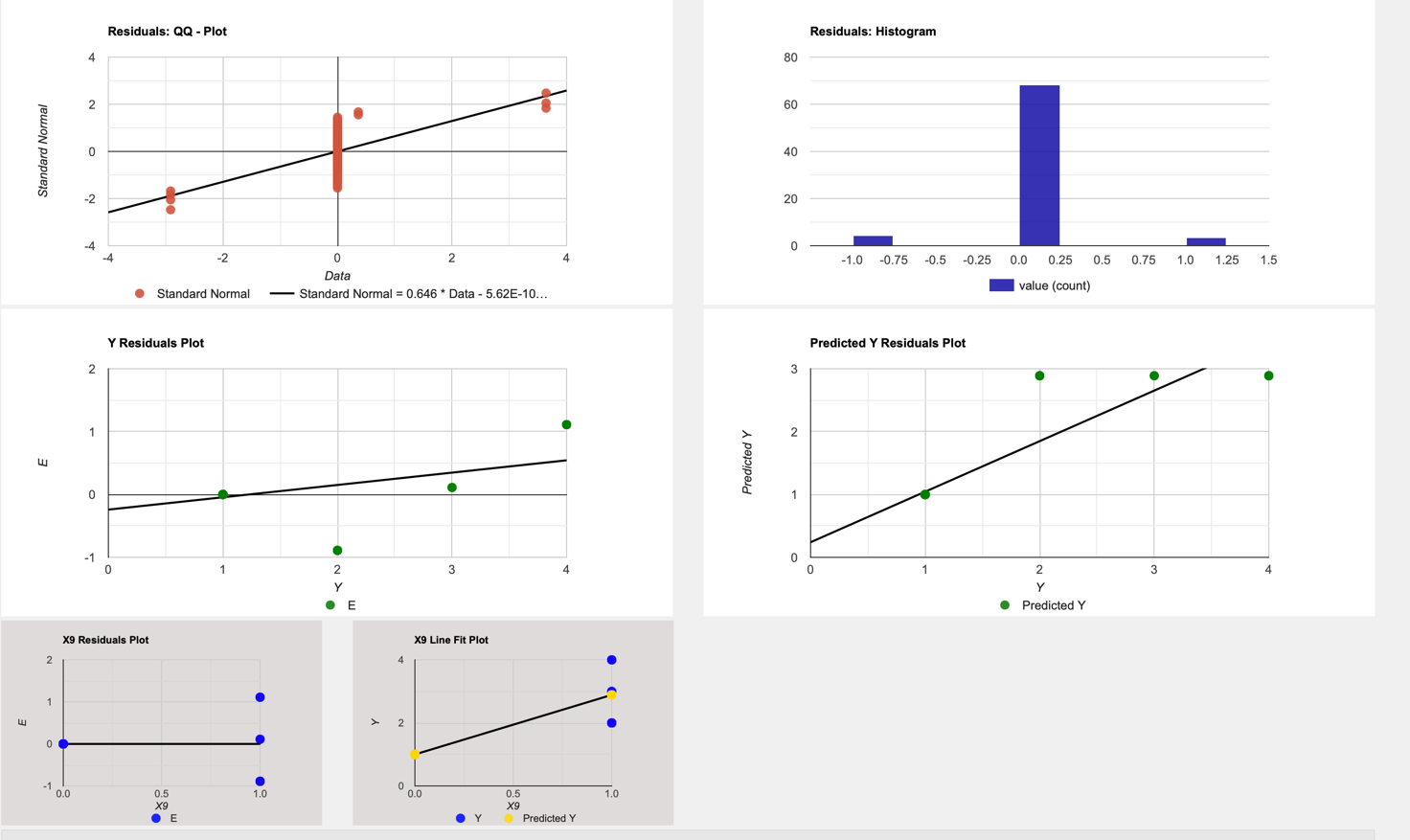


Effect of variables on the Months follow-up


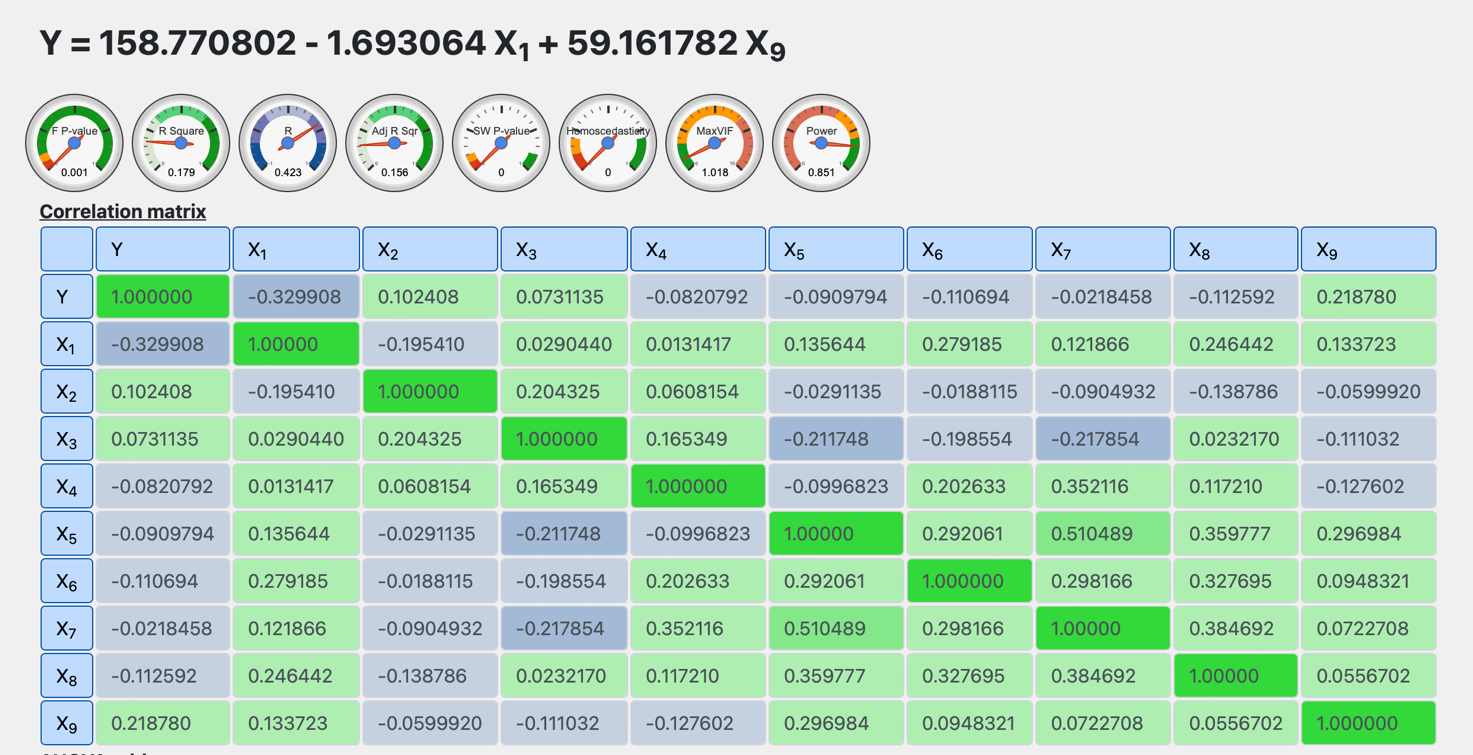


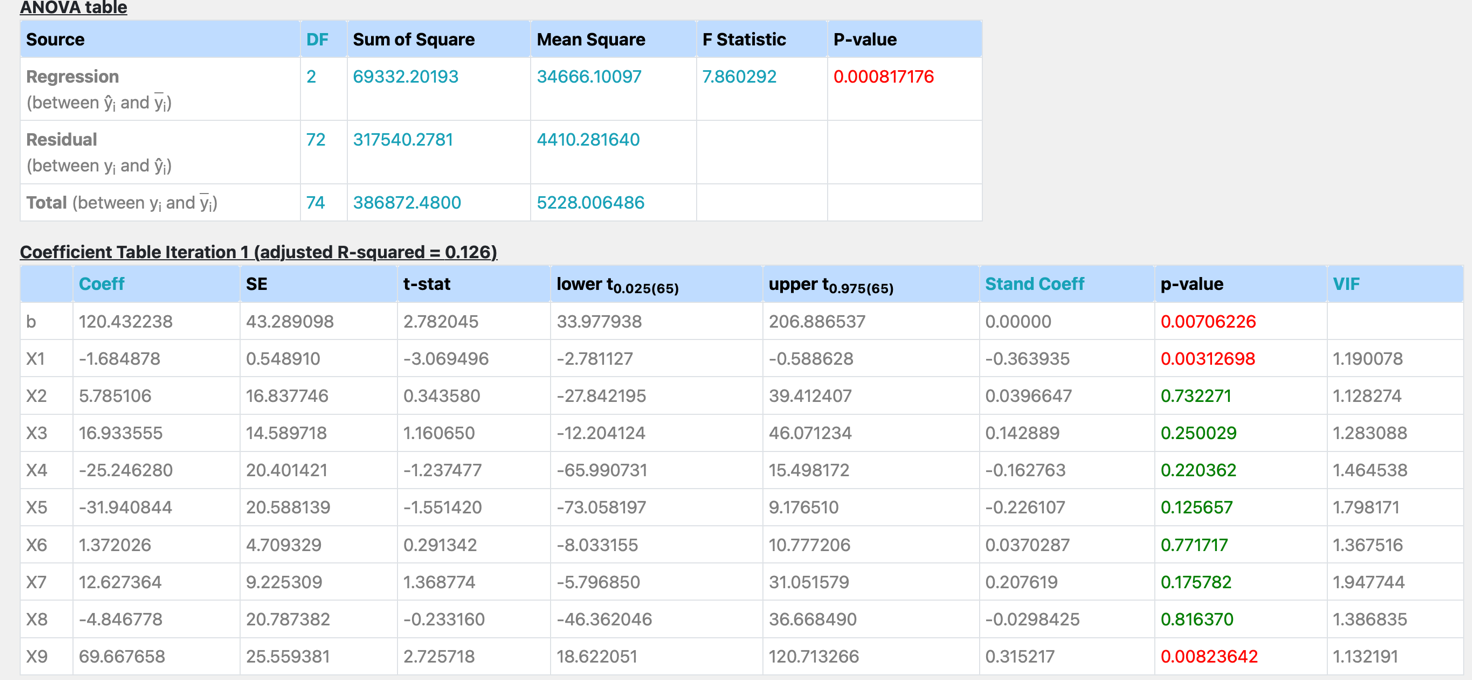


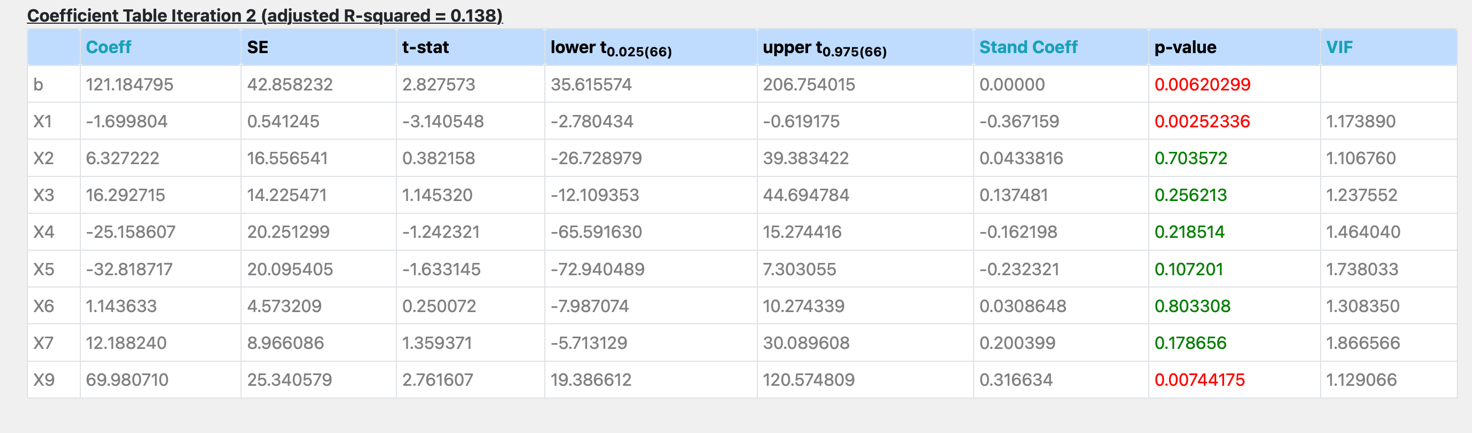


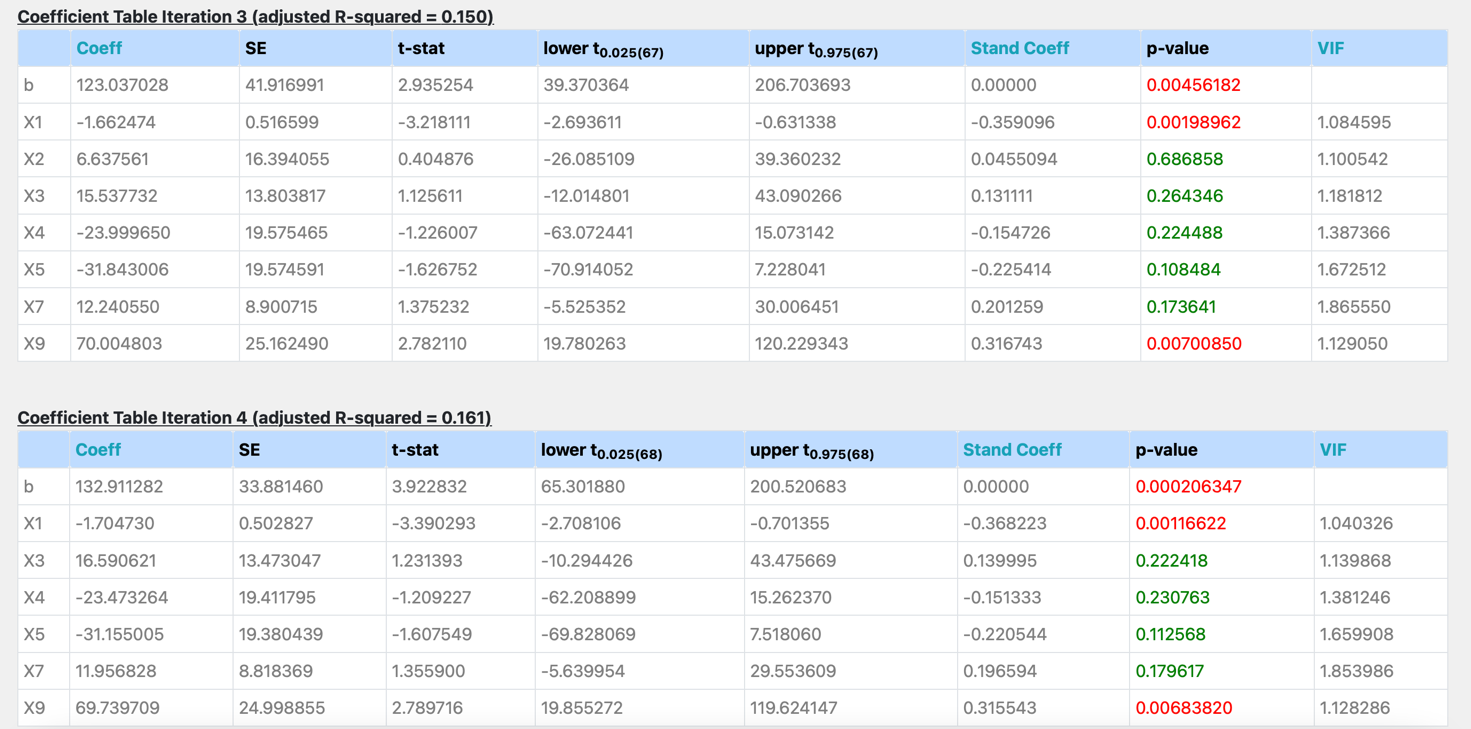


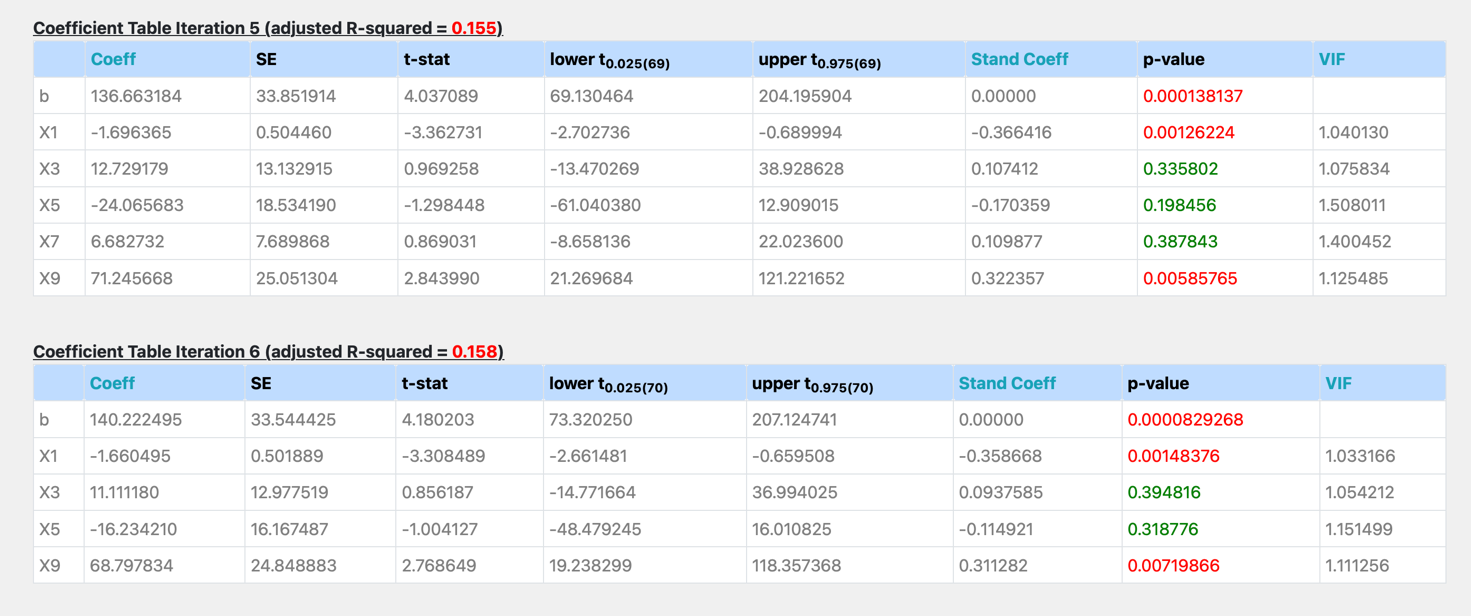

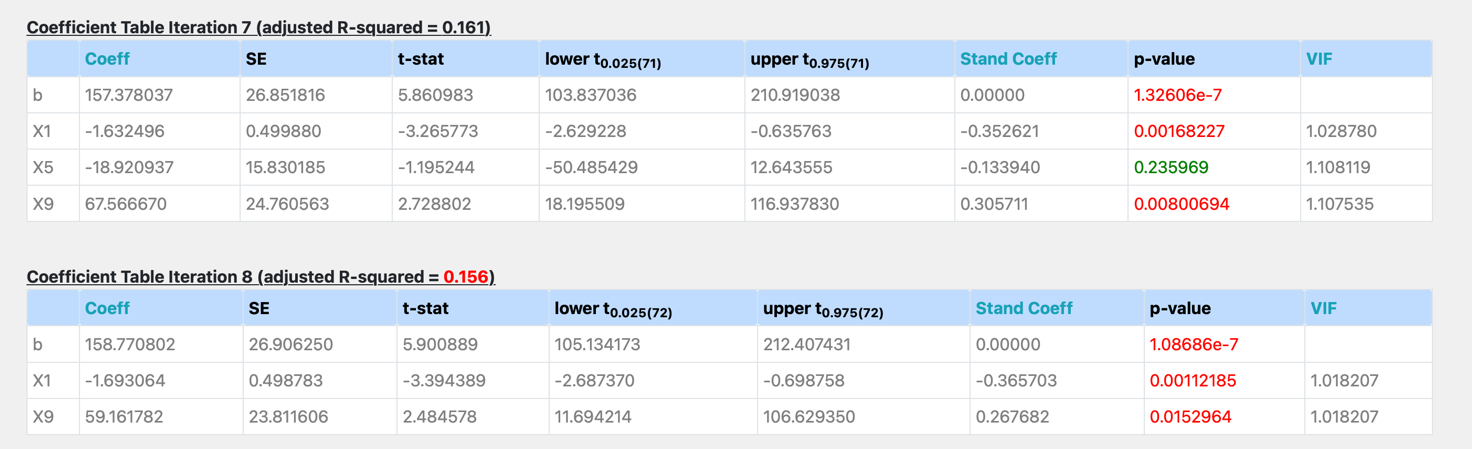


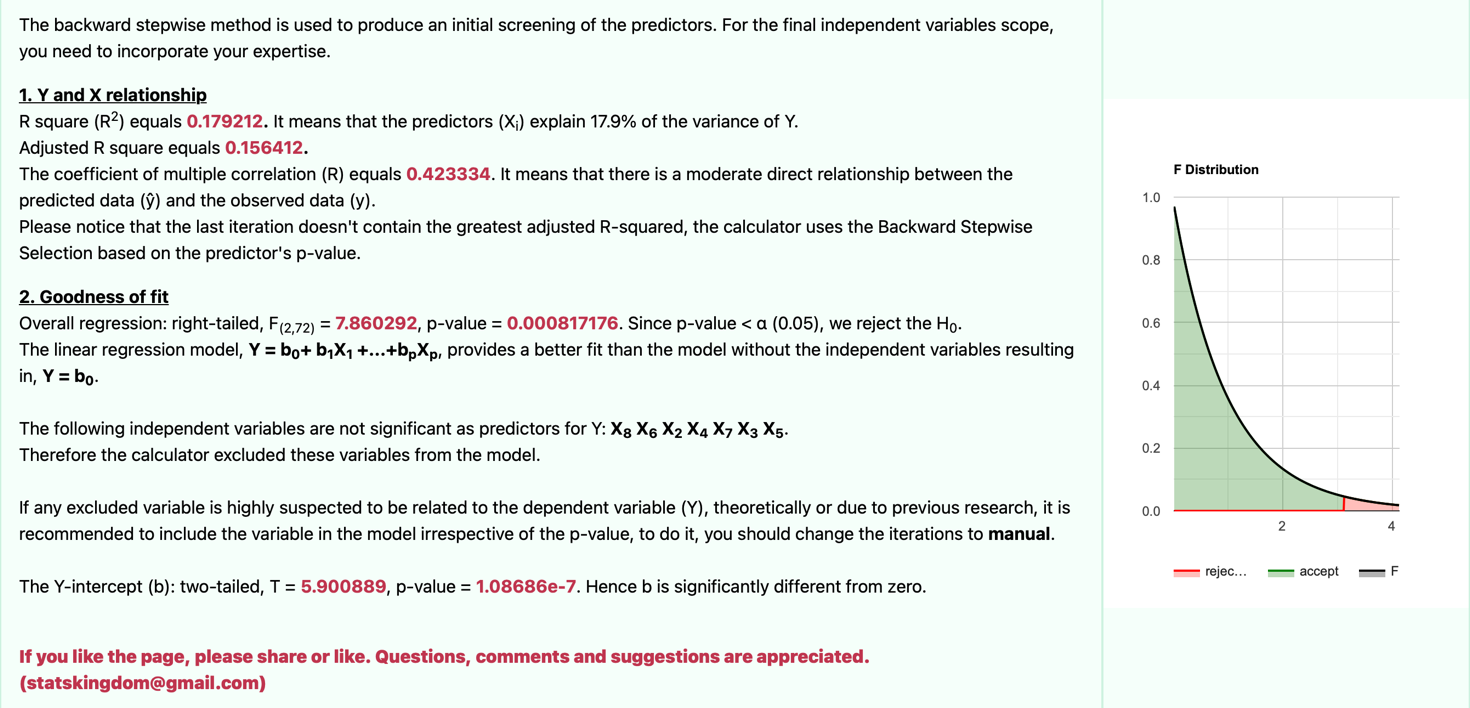


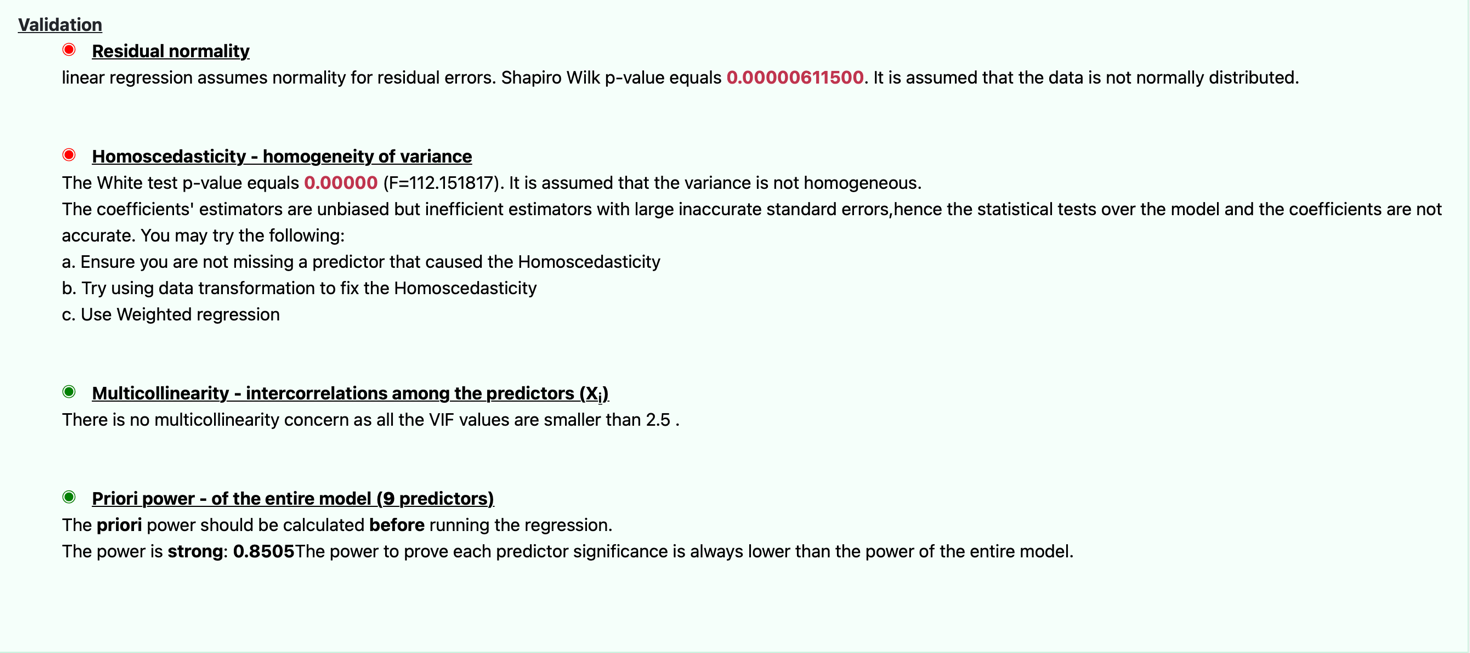


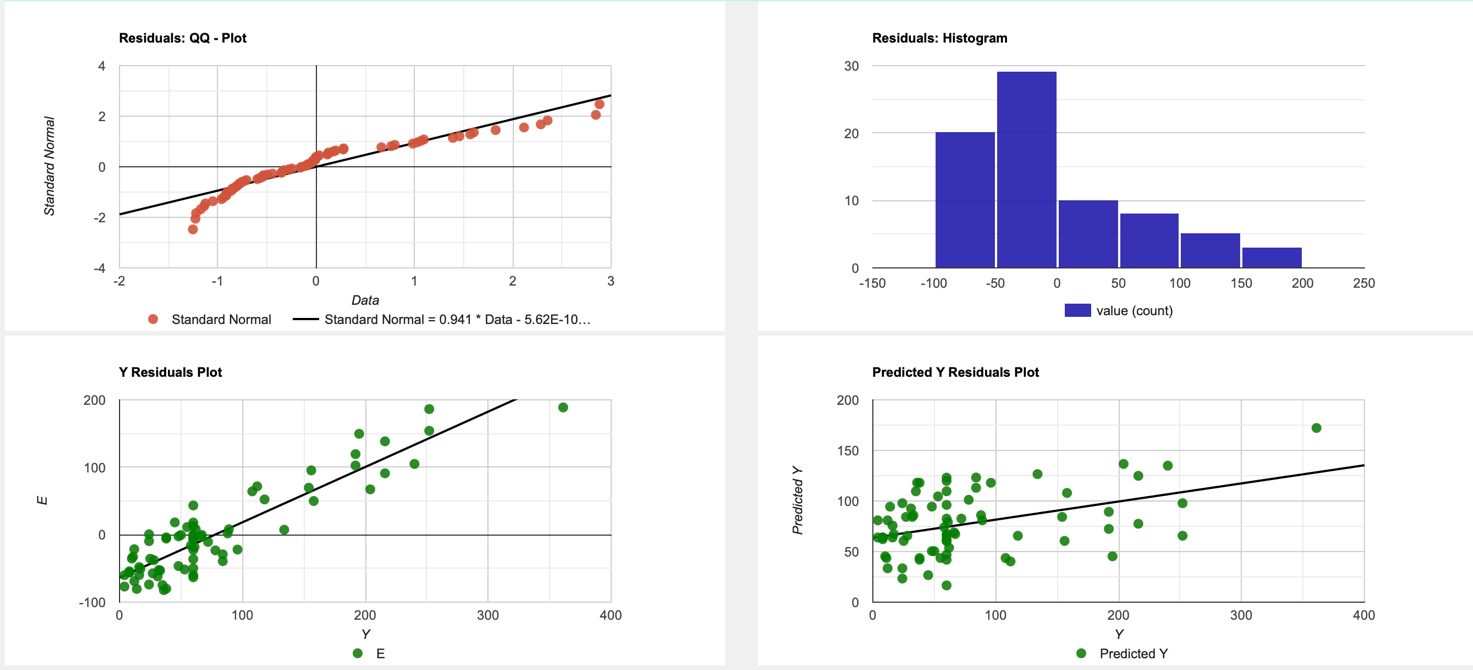


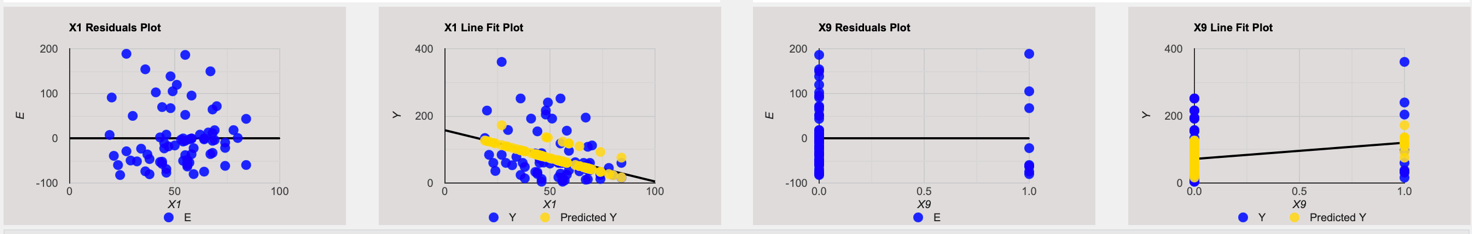

Supplement: Supplementary file 1 — Supplementary file1 (DOCX 5209 KB) [file 405_2022_7481_MOESM1_ESM.docx]
